# Moral imperative for immediate release of 2019-nCoV sequence data

**DOI:** 10.1093/nsr/nwaa030

**Published:** 2020-02-18

**Authors:** Chung-I Wu, Mu-ming Poo

**Affiliations:** 1 Section Editor for Life Sciences, NSR; 2 Executive Editor-in-Chief, NSR

Only two weeks ago, when the COVID-19 outbreak caused by the novel coronavirus, SARS-CoV-2 (previously named 2019-nCoV), started to look dangerous nationwide, we published an editorial in this space urging the instant posting of the population genomic data of the viruses [1]. Because these data are of grave significance to global human health, such an urgent call is justified and has been repeated elsewhere [2]. To our disappointment, the rate of releasing such data seems to have slowed down, when the need for them has become more acute.

We now repeat the call on a stronger ground of molecular evolution based on the preliminary analyses conducted by experts in the relevant fields (see Acknowledgements). Permissions to cite their results have been given. Seeing the emerging patterns, we now equate the immediate release with a moral imperative at a time of human suffering and social disturbances. We also fortify the proposed solution that encourages rapid dissemination of data, while discouraging unnecessary delay in their release.

## THE SCIENCE OF VIRAL EVOLUTION

The key rationale for the rapid data release is on account of a fundamental evolutionary principle that the viruses could be evolving rapidly after entering the human populations. Natural selection would select for mutations that drive efficient contagion that enhances their evolutionary advantages. A reduction in virulence may also accompany the increased contagion.

The evolutionary principle is corroborated by the lessons learned from the SARS outbreak [3]. During the period of 2002–03, the SARS virus spread slowly in the early stage (24 November 2002–30 January 2003). The spread accelerated quickly in the middle stage (February of 2003) and continued through the late stage for several more months. This speed-up is associated with changes in the viral DNA sequences. In particular, the S protein accrued five amino-acid (AA) mutations in quick succession in the early phase. In short, the SARS virus underwent several rounds of genetic adaptation after making the leap from the civet cats to humans.

The SARS outbreak, while informative, may or may not provide the right lesson for COVID-19. The quick release of the genomic information will enable the research community to compare the 2019-nCoVs with the SARS viruses for their evolutionary dynamics. Clinical reports have indicated substantial differences between the two episodes. Genomic analyses should therefore be employed now.

## A SMALL COMFORT—2019-nCoVs IN POSSIBLE EVOLUTIONARY STASIS

The preliminary analyses (Jie Cui and Jian Lu, personal communication) are based on the limited amount of data accessible to the public (https://www.gisaid.org/). As of 10 February 2020, there are 55 genomes of 2019-nCoVs available. The 31 sequences obtained before 22 January are almost entirely from within China (with one exception from the USA). Curiously, after 22 January, the representation flips completely, with all 24 sequences reported from outside China, including Japan, Korea, Singapore, Australia, the USA, France, England and others. Such partitions will make data interpretation difficult, as the influences of time and geography cannot be disentangled. This trend in data reporting (or acquisition) is troubling.

The preliminary analysis is nevertheless instructive by raising important questions. The key question is ‘Are 2019-nCoVs evolving adaptively in human populations?’ If the answer is no, then we should have already known much about 2019-nCoVs. It would be a small comfort if these viruses are not changing rapidly. At least in the period between December 2019 and early February 2020, this seemed to be the case. Among the 55 genomes analysed, only 8 AA mutations can be found in at least two samples. Most importantly, the distribution of these mutations (in technical terms, the frequency spectrum) is nearly the same as that of ‘silent’ mutations, which generally have little functional effects. This modest degree of evolution is in sharp contrast with the SARS virus at the stage of widespread infection.

In this scenario, 2019-nCoVs might have been fully evolved when they emerged from their secondary host into human populations. These viruses could have ‘cut their teeth’ as a lowly contagious virus between the secondary host and humans, becoming gradually adapted to humans in the process. The recent outbreak may have happened after the last step was taken. If pangolins are indeed the secondary host, as has been reported, then the small differences between the viruses in pangolins and humans might be this last step. The low evolutionary rate, the high contagious rate and the modest virulence all seem to suggest 2019-nCoVs to be in a mature stage of evolution in humans.

We hope this scenario will turn out to be true and the massive social anxiety can find a good reason to calm down. Precaution remains upmost but the uncertainty about the future would be a bit less worrisome.

## THE ALTERNATIVE POSSIBILITY OF A LOOMING CRISIS

While a possible evolutionary stasis is comforting, there are also disturbing signs. First, most of the AA changes discovered are from the more recent samples reported from outside China, thus hinting AA changes to be part of the viral adaptation. Second, the eight AA mutations appear clustered, as if having one AA mutation would make it more likely to acquire another one. Third, and particularly alarming, is the most successful AA mutation at position 28 144 of the ORF8 gene. This mutation occurs once in the 13 samples (7.8%) collected before 5 January, all from Wuhan. In the 42 samples collected after 10 January, all from outside Wuhan, the mutation is observed in 18 samples (43%). While this jump may seem startling, the sample size is really too small to afford strong statistical confidence. Hence, our call for more data is highly relevant here, as the 28 144 mutation may, or may not, be a very dangerous one. Furthermore, the second most successful AA mutation, although found in only 5 of the 55 samples, is scattered wide among samples from outside mainland China; they are from Orange County (CA2/2020, USA), Paris (IDF0373, 0373/2020, France), Kaohsiung (2/2020, China) and Clayton (VIC01/2020, Australia).

In reading the worst-case scenario, the overall patterns might suggest that 2019-nCoVs are about to make a move after spending 2 months in the slow-evolution mode. Two months is also roughly the length of the first stage of the SARS-virus evolution. We hope that the pattern observed above is due to stochastic fluctuations inherent in small data sets, especially the non-random ones (e.g. samples from the same family). There is no reason to be unduly alarmed but, again, accurate and timely releases of data remain the only way to be sure.

## THE CULTURE OF DATA (NON-)SHARING

When calling again and again for the immediate release of genomic data, we need to understand the cultural forces behind sharing vs. non-sharing of data. As stated above, the recent releases of data are mainly from outside China. The disputes about sequence release and utilization have been quite open on the internet and the root of the dispute is in securing credits. More specifically, it is about publication in a very small collection of journals with a high ‘Impact Factor’, or IFs [4]. The withholding is part of the publishing strategy.

While such a strategy is usually harmless to society, it would have devastating consequences in this current crisis. Indeed, the authors of the first viral sequences only revealed the information on human-to-human transmission in their formal publications, days after the disclosure by other public-health experts via alternative media. This possible delay will be pivotal in the future reviews of the unfortunate unfolding of the current event.

Underlying these irrational happenings is an almost pathological infatuation with publishing in selected journals, even though a paper published in either journal A or journal B is the same paper. Curing this pathological infatuation is probably most important to biological research in the long run [4].

## THE PROPOSED SOLUTION FOR FULFILLING THE MORAL OBLIGATION

Given the gravity of the current crisis, it would seem morally reprehensible to withhold data on the virus. Some websites have been created for posting such data (such as https://bigd.big.ac.cn/ or https://db.cngb.org or the Genowis data-analytic platform, fight-sars2.genowis.com). Academic journals, including *National Science Review*, must take a carrot-and-stick approach. On the carrot side, we have proposed that journals accept for publication genomic data lightly analysed soon after their acquisition [1]. To make the carrot attractive, we urge journals to accept the follow-up in-depth analyses by the sequence authors without demanding new data. Of course, this should only apply to the exigencies of an unusual time.

On the stick side, journals may need to consider the withholding of such data from public access unethical and ban the studies from publication. It is not difficult to determine the minimal time lag between obtaining genomic sequences and the completion of a manuscript. As the unethical treatment of experimental animals can result in the irrevocable rejection of a submission, the immorality of withholding vital data on human lives deserves no less attention.
